# Environmental Toxicants-Induced Immune Responses in the Olfactory Mucosa

**DOI:** 10.3389/fimmu.2016.00475

**Published:** 2016-11-04

**Authors:** Fumiaki Imamura, Sanae Hasegawa-Ishii

**Affiliations:** ^1^Department of Pharmacology, Penn State College of Medicine, Hershey, PA, USA

**Keywords:** olfactory epithelium, inflammation, immune system, intranasal administration, olfactory vector hypothesis, olfactory dysfunction, neurodegenerative disease

## Abstract

Olfactory sensory neurons (OSNs) are the receptor cells for the sense of smell. Although cell bodies are located in the olfactory mucosa (OM) of the nasal cavity, OSN axons directly project to the olfactory bulb (OB) that is a component of the central nervous system (CNS). Because of this direct and short connection from this peripheral tissue to the CNS, the olfactory system has attracted attention as a port-of-entry for environmental toxicants that may cause neurological dysfunction. Selected viruses can enter the OB *via* the OM and directly affect the CNS. On the other hand, environmental toxicants may induce inflammatory responses in the OM, including infiltration of immune cells and production of inflammatory cytokines. In addition, these inflammatory responses cause the loss of OSNs that are then replaced with newly generated OSNs that re-connect to the OB after inflammation has subsided. It is now known that immune cells and cytokines in the OM play important roles in both degeneration and regeneration of OSNs. Thus, the olfactory system is a unique neuroimmune interface where interaction between nervous and immune systems in the periphery significantly affects the structure, neuronal circuitry, and immunological status of the CNS. The mechanisms by which immune cells regulate OSN loss and the generation of new OSNs are, however, largely unknown. To help develop a better understanding of the mechanisms involved, we have provided a review of key research that has investigated how the immune response in the OM affects the pathophysiology of OSNs.

## Introduction

We are continuously exposed to a variety of potentially harmful environmental agents, such as bacteria, viruses, mold, dust, pollen, and environmental chemicals. Environmental agents entering the nasal cavity may become allergens, causing inflammation in the olfactory mucosa (OM) (olfactory inflammation), and leading to allergic rhinitis and infectious sinusitis (1). The symptoms are usually associated with hyposmia or anosmia (2, 3). Olfactory loss in rhinitis/sinusitis is attributable primarily to blockade of airflow to the olfactory sensory neurons (OSNs) that receive odorous molecules, but damage to the OM is also considered as a possible cause (2, 4–6). In fact, multiple studies have shown that olfactory inflammation causes the loss of OSNs (7–14).

Epidemiological studies have associated exposure to environment toxicants with the incidence of neurodegenerative diseases, including Alzheimer’s and Parkinson’s diseases (15, 16). Since olfactory dysfunction is a common prodromal symptom of these diseases, and because xenobiotics administered into the nasal cavity are often found in the brain, the nasopharynx has attracted attention as a port-of-entry for environmental agents that cause neurological disease (the olfactory vector hypothesis) (17–19). To date, a variety of neurotoxicants have been directly administered to the naris of model animals to study their transport to the brain and the resultant neurodegenerative effects in the central nervous system (CNS). Results of these types of studies have now been summarized in a number of reviews (17, 20–22).

Neuroinflammation is a hallmark of neurodegenerative diseases (23–26). Although knowledge of the cause of neuroinflammation is still limited, olfactory inflammation has been proposed as one of the major mechanisms (27, 28). Interestingly, allergic rhinitis is associated with development of Parkinson’s disease later in life (29). We, therefore, believe that a better understanding of the olfactory immune system will advance our knowledge of the pathogenesis and progression of neurological disease. To date, there are many reports showing that artificially induced olfactory inflammation can cause immune responses and damage to the OM (7–14). Conversely, new OSNs are generated in the OM throughout life, which may help in the repair of damaged tissue (30, 31). It also has been reported that immune cells in the OM regulate the depletion of old OSNs and generation of new OSNs. This review summarizes the roles of immune cells in the inflammatory response, tissue damage, and regeneration of the OM with a focus on model systems, primarily the OM of murine species.

### Structure of the Olfactory Mucosa

**Figure 1:**
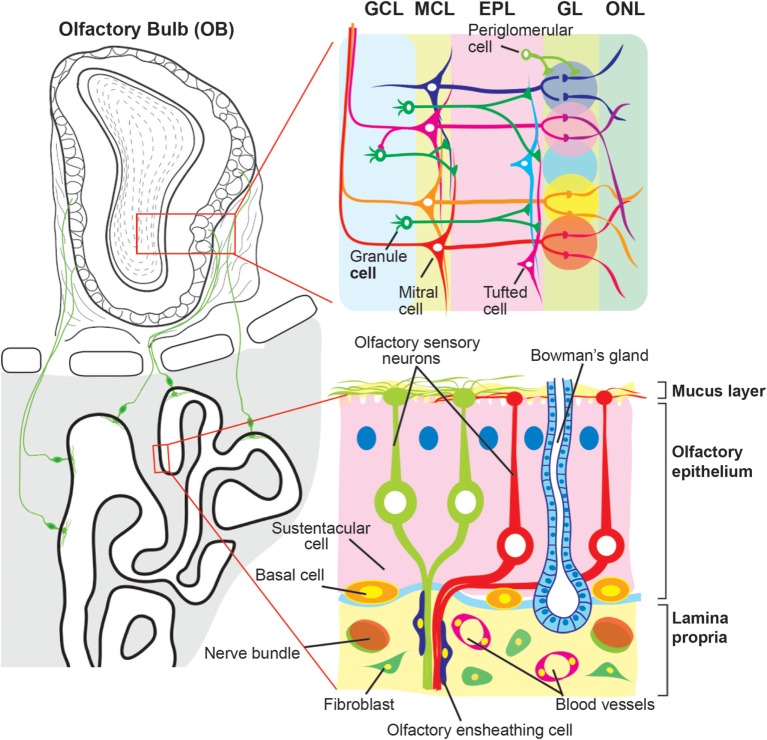
**Schematic diagram of the olfactory mucosa and olfactory bulb**. The olfactory mucosa is composed of the olfactory epithelium (OE) and the lamina propria. Three types of cells are found in the OE, olfactory sensory neurons (OSNs), sustentacular cells, and basal cells. The dendrites of OSNs project toward the mucus layer where they protrude the cilia expressing odorant receptors. Sustentacular and basal cells are localized at the apical and basal regions of the OE, respectively. The lamina propria is a layer of connective tissue, which lies beneath the OE, and contains fibroblast, blood vessels, and Bowman’s gland. OSN axons are fasciculated into bundles that are wrapped with olfactory ensheathing cells and target the olfactory bulb (OB) by passing through the lamina propria and cribriform plate. The OB is divided into multiple layers. Each OSN axon runs the surface of the OB, olfactory nerve layer (ONL), and projects to a glomerulus in the glomerular layer (GL). There, the OSN axons synapse with primary dendrites of projection neurons, mitral and tufted cells, and onto populations of interneurons, periglomerular cells. The secondary dendrites of mitral/tufted cells make dendrodendritic synapses with another population of interneurons, granule cells, in the external plexiform layer (EPL). Beneath the EPL, the OB has the mitral cell layer (MCL) and granule cell layer (GCL) where the cell bodies of mitral cells and granule cells are located, respectively.

The OM is located in the upper region of the nasal cavity, and is made up of the olfactory epithelium (OE) and the underlying lamina propria (Figure 1). The surface of the OE is covered with a mucus layer where inhaled odorant molecules can be trapped, which then bind to odorant receptors expressed on the cilia of the OSNs whose cell bodies are located in the OE. Unlike other receptor cells, OSNs project directly to the olfactory bulb (OB), the first relay station of olfactory information in the CNS, through the cribriform plate. Sustentacular cells line the apical surface of the OE, and provide trophic, metabolic, and mechanical support for OSNs. At the basal surface of the OE, there are two types of basal cells (horizontal and globose basal cells) that give rise to new OSNs and sustentacular cells during lifetime of the organism.

The lamina propria is a layer of connective tissue through which OSN axons pass. OSN axons, although unmyelinated, are gathered into bundles (olfactory nerve) that are wrapped with olfactory ensheathing cells (OECs). OECs are specialized glial cells that are resident in the olfactory system. The lamina propria also contains Bowman’s glands and vascular elements. Bowman’s glands produce mucus and secrete it to the mucus layer *via* the OE duct.

### Response of Olfactory Mucosa to Intranasal Administration of Environmental Agents

Inhalation of harmful environmental agents often damages the OM. Here, we focus on the damages associated with inflammatory responses within the nasal cavity (32). Several animal models of human chronic rhinosinusitis have been developed by inoculating bacteria or fungus extract into the mouse nostril (10, 33, 34). These mouse models have shown inflammatory responses in the nasal cavity, as well as general pathology of the OE that includes mast cell and eosinophilic infiltration into the respiratory epithelium, with increased depth of lamina propria. In addition, olfactory inflammation can be caused by a single compound derived from microbial pathogens, such as polyinosinic:polycytidylic acid [Poly(I:C)] (14), lipopolysaccharide (LPS) (9), satratoxin G (SG), and roridin A (RA) (7, 8, 11, 13). Poly(I:C) is a synthetic analog of viral double-stranded RNA, and is recognized by Toll-like receptor 3 (TLR3) (35), whereas LPS is an endotoxin found in the outer membrane of Gram-negative bacteria that activates another type of Toll-like receptor, TLR4 (36, 37). When injected intraperitoneally, LPS caused systemic inflammation that also changes the level of inflammatory cytokines in the brain (38). SG and RA are macrocyclic trichothecen mycotoxins produced by fungi such as *Stachybotrys chartarum*, the “black mold” (39). Immunohistochemical analyses using TLR3 and TLR4 antibodies indicated that sustentacular cells and OECs may be the first target cells of PolyI:C and LPS in the OM, respectively (14, 40). Besides activating different receptors, therefore different types of cells, intranasal inoculation of each of these agents causes an inflammatory response and damage to the OM of rodents. It is useful to review what is known about intranasal inoculation of Poly(I:C), LPS, SG, and RA and their effects on olfactory tissues.

### Inflammatory Response

Infiltration of neutrophils expressing Ly-6G/-6C into the OM occurs 1 day after intranasal inoculation of Poly(I:C), SG, or RA (7, 8, 14). Kanaya et al. confirmed that Poly(I:C) caused the infiltration of macrophages (F4/80+) and T-lymphocytes (CD3+) (14). In contrast to the situation with neutrophils, which completely disappeared within 6 days, significantly higher numbers of macrophages and T-lymphocytes were observed in the OM as long as 21 days after the last Poly(I:C) inoculation. In addition, in the OM, Poly(I:C), SG, or RA caused upregulation of mRNAs encoding inflammatory cytokines, including IL-1α, IL-1β, IL-6, TNF-α, and MIP-2.

### Damage of the Olfactory Mucosa

Intranasal inoculation of environmental agent-derived components also damaged the OM and led to apoptosis of OSNs and decreased thickness of the OE (7–9, 14). When Poly(I:C) was inoculated into mouse nostril once a day for 3 days, the number of apoptotic cells was significantly increased and the number of OSNs was decreased in first 3 days. When examined 9 days after the first inoculation (i.e., 6 days after the last inoculation), few apoptotic cells were observed in the OE, but the number of OSNs was less than that observed 3 days post inoculation.

The mechanisms underlying OSN loss associated with olfactory inflammation are currently not well understood. Inflammatory responses seem to play a critical role for death of OSNs. During inflammation, neutrophils and macrophages secrete elastases, proteases known to break down bacterial membrane proteins (41). Intranasal inoculation of neutrophil elastase caused the loss of OSNs in the OE (14). In contrast, Poly(I:C)-induced damage of the OM was blocked by prior intraperitoneal injection of the neutrophil elastase inhibitor, Silevestat (14). In addition, inflammatory cytokines are also involved in inflammation-induced OSN death. Lane and colleagues created a transgenic, induced olfactory inflammation (IOI) mouse model, in which expression of TNF-α in sustentacular cells was induced with doxycycline as a chronic rhinitis model (12). Using this transgenic mouse, Lane et al. showed that induction of TNF-α expression caused marked reduction of OE thickness and loss of OSNs, whereas the sustentacular cells were unaffected. Concurrent treatment with prednisolone (to inhibit downstream inflammatory responses) prevented OSN loss, therefore suggesting that TNF-α does not directly cause OSN apoptosis (42).

The damage to the OM exacerbates the impact caused by exposure to environmental toxicants. While *Staphylococcus aureus* is an indigenous microbe found in the nose and usually remains in the lumen after intranasal administration, bacteria can still penetrate the OE and cause an inflammatory response when inoculated intranasally after first damaging the OM with Triton X-100 or zinc sulfate (43). In addition, tissue damage coupled with an inflammatory response of the OM induced by RA was exacerbated by co-exposure to LPS (8). Although mRNAs encoding inflammatory cytokines were marginally induced by RA alone, co-exposure to both RA and LPS dramatically elevated expression of these genes.

### Regeneration of OE

Basal cells of the OE give rise to new OSNs and sustentacular cells throughout life (44, 45). Damage of basal cells caused by methyl bromide gas, however, led to the eventual loss of OSNs and inhibited the reconstruction of OE (46). In contrast, these basal cells seem to be less affected by intranasal inoculation of Poly(I:C), LPS, or mycotoxins; apoptosis was restricted to OSNs whose cell bodies reside in the middle layer of the OE, below the apical row of supporting cell nuclei and above the basal cell nuclei (7, 8, 14). Cells expressing Ki67, a proliferating basal cell marker, were increased in number and distributed in all layers of the OE 6 days after the last Poly(I:C) inoculation (14). The OE thickness and the OSN number were almost completely recovered by 21 days post-Poly(I:C) exposure. However, the recovery of OSNs was incomplete (40–50%), even at 21–28 days after the last inoculation of SG (7, 11).

Immune cells and OECs in the OM also regulate many aspects of degeneration/regeneration of OSNs. The resident macrophages play a key role in the removal of cell debris and stimulation of basal cells to proliferate (47). OEC is known to be a specialized type of glia that wraps OSN axons, as well as serves as a major phagocytic cell type (48, 49). Bulbectomy or olfactory nerve transection causes apoptotic death of OSNs by severing the axons and stimulates the generation of new OSNs (50–53). Bulbectomy activates the proliferation of OECs in lamina propria (54) and the infiltration of macrophages into the OM (55). In addition to the phagocyotosis of apoptotic cellular debris, infiltrated macrophages secrete a variety of inflammatory cytokines and chemokines, such as LIF, IL-6, MCP-1, and MIP-1α (52, 56, 57). It has been proposed that MCP-1 and MIP-1α play key roles in recruitment of additional macrophages to the OM and that LIF stimulates globose basal cells expressing LIF receptor (LIFR) (52, 57, 58). Activation of LIFR subsequently induces iNOS expression that in turn stimulates proliferation of neural precursor cells (59). An increase in iNOS level was also observed in OECs after bacterial challenge to the compromised OM (60).

## Discussion

This review has summarized olfactory inflammation caused by intranasal inoculation of Poly(I:C), LPS, and mycotoxin. Although the receptors and signaling pathways activated by these agents are not identical, they induce similar effects on the OM, including infiltration of immune cells, upregulation of inflammatory cytokines, and loss of OSNs. It appears that damaged and lost OSNs can be replaced with new OSNs since olfactory inflammation has minimal effect on the basal cells. It is not clear, however, whether basal cells are, in fact, affected by olfactory inflammation. The inoculation of toxicants into the IOI-transgenic mouse showed that TNF-α-induced inflammation lasted for 6 weeks and compromised the regeneration of OSNs, although the effect was not permanent, suggesting that TNF-α suppresses the proliferative activity of basal cells (12, 61). In contrast, intraperitoneal injection of the herbicide 2,6-dichlorobenzonitrile induced inflammation-like pathological changes in OE and depleted the horizontal basal cells, resulting in permanent loss of OSNs (62). A critical next step is to elucidate the molecular mechanisms underlying the specific loss of OSNs and the resistance of basal and sustentacular cells to olfactory inflammation. Since variety of immune cells are involved in inflammatory responses, detailed researches on types of immune cells activated and infiltrated in the OM during olfactory inflammation are required to elucidate the mechanisms.

It is also known that zinc sulfide and hydrogen sulfide administered into the nasal cavity induces the loss of OSNs (63–67), and anosmia induced by intranasal zinc has been suggested to occur in humans (68, 69). The immune response caused by exposure of the nasal cavity to toxic gases and metals is not well understood, but the regions of the OM affected by hydrogen sulfide inhalation is different from the regions affected by either intranasal Poly(I:C), LPS, or by mycotoxin inoculation. The OE can be subdivided into several zones based on the expression patterns of specific molecules (including olfactory receptors), and the dorsal medial meatus largely overlaps with zone 1 (aka dorsal zone) (70). Inhalation of hydrogen sulfide provoked necrotizing lesions of the OSNs predominantly localized in the zone 1 (64, 65), whereas the OE lining the dorsal medial meatus was not affected by the intranasal inoculation of Poly(I:C), SG, or RA solution (7, 11, 14). The difference in susceptible portions in the OE may be attributed to the different flows of liquid and gas in the nasal cavity (71). Alternatively, molecules exclusively expressed by OSNs in zone 1 (e.g., NQO1, O-MACS, and Dvl-1) or in zone 2–4 (e.g., OCAM and Foxg1) may determine the susceptibility of OSNs to the environmental agents (72–76). Understanding the similarities and difference in immune responses to different environmental agents will help us to evaluate the risks to the CNS.

According to the olfactory vector hypothesis, some neurological disorders are caused or accelerated by agents entering the OB *via* the OM (17). Of interest, it was shown that either intranasally administered influenza virus, LPS, or MPTP (a synthetic neurotoxicant) caused selective decreases of dopamine neurons in the substantia nigra of mice (21, 27, 77–79). The route from the OM to the substantia nigra, however, remains to be elucidated. The transport of viruses, bacteria, and metals from the nasal cavity to the OB has been reviewed by others (17, 20–22). Interestingly, Nipah virus propagates anterogradely in the hamster CNS *via* the olfactory pathway beginning in the OB (80). The agents entering the OB may spread further in the brain to cause neurological disorders. It is suggested that inflammatory responses can spread in the CNS both anterogradely or retrogradely *via* axonal projections (81). For instance, corneal inflammation induced by instillation of benzalkonium chloride damages primary sensory neurons in the trigeminal ganglion, leading to the activation of second-order neurons and glial cells in the brain stem and to the production of pro-inflammatory cytokines (82). Therefore, the inflammatory response may propagate in the brain from the primary olfactory tissue. Although this review focused on the effects on the OM, olfactory inflammation was also associated with atrophy of the OB; upregulation of the mRNA levels of inflammatory cytokines; infiltration of neutrophils; and/or activation of astrocytes and microglia (7, 8, 11, 43). These changes clearly should affect the OB neurons. Furthermore, intranasal LPS injection caused upregulation of TLR2 signals in the OB, which spread to other parts of the brain within 24 h (83). Further studies of neuroinflammation and damage in other brain regions will provide us with novel insights into the olfactory vector hypothesis and the pathogenesis of neurological disorders.

## Conclusion

The olfactory system is a unique site where the peripheral nervous system and CNS are in close proximity. Since the OM is bathed in a sustained exposure of environmental agents that may cause inflammatory responses, the health of the CNS is likely to be heavily influenced by the immune status of the olfactory system. Big challenges in future are (1) to determine whether olfactory inflammation contribute to pathogenesis of neurodegenerative diseases; and (2) to determine whether olfactory inflammation sequentially affect the immune status of the CNS *via* the olfactory pathways in the brain.

## Author Contributions

FI and SH-I searched and reviewed previous works and wrote this article.

## Conflict of Interest Statement

The authors declare that the research was conducted in the absence of any commercial or financial relationships that could be construed as a potential conflict of interest.
